# Study protocol of the ESUB-MG cluster randomized trial: a pragmatic trial assessing the implementation of urine drug screening in general practice for buprenorphine maintained patients

**DOI:** 10.1186/s12875-016-0413-3

**Published:** 2016-03-01

**Authors:** The ESUB-MG Study Group

**Affiliations:** Département Universitaire de Médecine Générale, Université de Toulouse III, Faculté de Médecine, 133 route de Narbonne, 31062 Toulouse, France

**Keywords:** Opioid related disorder, Opiate substitutive treatment, Buprenorphine, Substance abuse detection, Urine drug screening test, Outpatient, General practitioner, Maintenance

## Abstract

**Background:**

In addiction care, urine drug screening tests are recommended to assess psychoactive substances use. While intrinsic diagnostic value of these tests is demonstrated, the consequences of carrying out these tests on opiate maintenance treatment (OMT) have not been established. The main objective will be to assess the impact of on-site urine drug screening tests (OS-UDS) in general practice compared to routine medical care on OMT retention at 6 months in opioid-dependent patients initiating buprenorphine.

**Methods/Design:**

The ESUB-MG study uses a pragmatic, cluster randomized controlled trial design. General Practitioners (GPs) regularly managing patients treated with buprenorphine and consenting for participating will be invited to participate. GPs will be randomly assigned to one of two groups for 6 to 24 months: (a) control group (usual care: standard medical strategy for assessing drug use); (b) interventional group (including 1/ a training session on practice and interpretation of OS-UDS; 2/ the supply of OS-UDS at GPs’ medical offices; 3/ performing an OS-UDS before the first prescription of buprénorphine). GPs will have to include 1 to 10 patients aged 18 years-old or more, consulting for starting treatment by buprenorphine, not opposed to participate. The primary outcome will be OMT retention at 6 months.

**Discussion:**

This randomized interventional trial should bring sufficient level of evidence to assess effectiveness of performing OS-UDS in general practice for patients treated by buprenorphine. Training GPs to drug tests and supplying them in their office should lead to an improvement of opioid-addicted patients’ care through helping decision.

**Trials registration:**

NCT02345655 (first registration May 14, 2014)

## Background

Opioid addiction is currently defined as a “chronic, relapsing disorder” [1, 2]. Mortality of untreated heroin dependence is consistently estimated at 1–3 % per year, at least half of which is because of heroin overdose [3]. Beyond mortality and morbidity, heroin dependence inflicts enormous social and economic costs due to crime, unemployment, relationship breakdown, and the cost of law enforcement. Data from systematic reviews show that methadone maintenance is the most effective treatment in retaining patients in treatment and suppressing heroin use [4, 5]. The systematic review by Mattick et al. demonstrated the efficacy of buprenorphine maintenance treatment, with a lower retention rate than methadone but giving a similar decrease in opiate consumption [5]. The combination of buprenorphine and naloxone (marketed as Suboxone®) was created to prevent the injection of buprenorphine. Nevertheless, its effectiveness in preventing intravenous use of buprenorphine is not yet clear [6, 7].

The management of opiate-dependent patients in France is shared among specialized centres and general practitioners (GPs). Three drugs are available for the maintenance treatment methadone, buprenorphine (approved since 1995) and buprenorphine-naloxone (approved since 2006). Methadone must be initially prescribed by practitioners in specialized addiction centers, whereas buprenorphine and buprenorphine-naloxone should be prescribed by any general practitioner and available through community pharmacies. The number of people receiving maintenance treatment was estimated around 100,000 for high-dose buprenorphine and around 40,000 for methadone in 2010 [8]. Because the availability and the easy access to buprenorphine [9] most of opiate-dependent patients are managed in general practice [10]. Actually, few GPs take care of these patients: they were only 24 % in 2009 to regularly take care of patients under opiate maintenance treatment (OMT) [11]. One of their difficulties is to evaluate the concordance between the patient’s word and his drug use (OMT and other illicit drugs) [12] while associated drug use is frequent (at least one or two thirds of patients under OMT would also consume alcohol and benzodiazepines [13]).

In the field of addiction, assessment of substances consumption is crucial for diagnosis and, even more, for medical management. Nevertheless, this assessment is often difficult as self-reports use to under estimate psychoactive substances’ consumption [14–18]. One Scandinavian study in 2009 [19] compared the assessment by the psychiatrist of drug consumption in an emergency setting to that obtained by a urine drug screening test (UDT); the reference method being chromatography-tandem mass spectrometry. The physician had correctly assessed the consumption of alcohol but the assessment for illicit substances was not so good for other- psychoactive substances. The value of urine drug screening was assessed comparing urine screening tests’ results and chromatography. At the same time, urine drug screening presented a better sensitivity for benzodiazepines, opiates and cannabis. This study clearly showed that physicians’ assessment of psychoactive drug use lead to underestimate the true consumptions, and UDT would be useful in making decisions about treatment.

By contrast, in certain context, patients can overestimate their use of psychoactive substances notably when entering a detoxification program [20].

French guidelines highlight the need to assess opiate dependence for the management of patients taking OMT: screening tests have to be performed before introducing methadone; they are recommended before starting treatment with buprenorphine and during the follow up of both treatments [21]. Methadone prescription guidelines detail the recommended urine tests: a first, mandatory test before starting methadone treatment and later control tests. The first urine test confirms current drug consumption and the absence of methadone intake. Tests are subsequently done once or twice a week during the first 3 months of treatment, then twice monthly. When the patient has transferred to an outpatient setting, tests can be done if the physician considers it necessary. Tests are not obligatory for buprenorphine, but highly recommended. In 2011, French guidelines advised a standardized screening test schedule in the initiation and follow-up of buprenorphine treatment [22].

The drug tests can be carried out by immunochemical methods, either by automated analyzers in the biology laboratory or by drug screening kits. These tests (whether on a laboratory automat or using a commercial kit) are qualitative and have defined thresholds. False negatives exist for cocaine and benzodiazepines in particular, but test quality is intrinsically better for opiate detection (1.9 % false negatives) [23]. Results can be confirmed by the reference method, liquid or gas phase chromatography with mass spectrometry, which gives quantitative measurements [24]. Screening can be done during the patient’s visit, in either a specialized addiction centre or the physician’s office. Laboratory tests, regardless of the method or the biological medium, are reimbursed by the French health insurance system with no limit on their number or time period. In medical offices, immunoassays have been shown to be reliable [25, 26]; nevertheless, on-site urine drug screening (OS-UDS) by commercial kits are not reimbursed by the French health insurance system but some specialized addiction networks in France provide them to their members.

In a recent study performed among a sample of French GPs, only 12.2 % of GPs reported to perform urinary screening tests and in this proportion were counted GPs who reported to use them for other reasons than initiation and follow up of OMT [11]. The main reason for not performing UDS was a lack of knowledge about screening tests [11]. Among the few GPs using tests, the consequence they reported was mainly reinforcing dialogue with the patient. In another study performed in the same area among 1,507 patients initiating an OMT with buprenorphine or methadone, only 2.6 % had at least a drug test reimbursed by the Health Insurance System during their addiction treatment period [27].

Few studies explored the consequences of carrying out these tests on medical management. In the previous cited study, having been drug tested was associated with a better opiate substitution maintenance, with 45 % decrease of drop-outs (95 % CI: 0.38–0.80) [27]. In a retrospective cohort study of methadone users performed through data obtained from a primary care prescription registry in Tayside, Scotland, a history of having urine tested was a protective factor in relation to all-cause mortality with a reduction of 70 % of risk of death (HR 0.33, 0.22 to 0.49) [28]. On the basis of the literature, one would suppose that carrying out UDS would provide an improvement in the management of patients with opioid addiction and positive outcomes for patients such as longer opiate substitution maintenance and its clinical consequences in which a decrease in mortality. However, this hypothesis relies only on observational data, and we cannot rule out confusion bias.

In order to provide more consolidated data on the interest of using UDT in the context of general practice, we propose an interventional trial, which the main objective is to assess the impact of on-site urine drug screening tests in general practice compared to routine medical care on OMT retention at 6 months in opioid-dependent patients initiating buprenorphine. Second objectives are to assess the acceptability of OS-UDS by patients and GPs, to assess patient adherence to buprenorphine and to assess associated psychoactive consumptions.

## Methods/Design

### Study design

The ESUB-MG study is a pragmatic, cluster randomized controlled trial design. Clustering is at the level of the GP. A cluster design is needed as the intervention concerns the GP and as the evaluation concerns the patient. Furthermore, patients of a same GP are more correlated than patients of different GPs.

### Research objectives

This study is designed to assess the impact of on-site urine drug screening tests in general practice compared to routine medical care on OMT retention at 6 months in opioid-dependent patients initiating buprenorphine.

### Study population

#### GP inclusion criteria

Professional criteria: to practice as a GP, to be in activity, to practice in general ambulatory practice (in a medical office).

Patients’ characteristics: to regularly manage patients treated with buprenorphine.

Legal characteristic: to be registered in sector 1 (a registered doctor has an agreement with Social Security and the fees he applies correspond to ‘reasonable and tactful’ tariffs set by the social security system, whereas a non-registered doctor is authorised to charge higher fees. A sector 1 registered general practitioner charges no more than the statutory fee).

Consent for participating to the trial.

#### GPs exclusion criteria

To practice in a group medical office in which another GP has been included in the trial.

#### Patients' inclusion criteria

Aged 18 years-old or more.

To consult for starting buprenorphine or buprenorphine-naloxone (as several periods of OMT exist for a same period, a period of more than 6 months without OMT will be sufficient to consider that a patient starts buprenorphine or buprenorphine-naloxone).

Affiliated to a health insurance scheme.

Not opposed to participate.

#### Patients’ exclusion criteria

- To consult for continuing buprenorphine or for another complain related to opiate substitution treatment.
- To be known and yet managed by the GP for an opiate maintenance treatment.
- To have started buprenorphine or buprenorphine-naloxone in a specialized centre or in a hospital.
- To be treated with methadone.
- To be treated with methadone and asking a switch toward buprenorphine or buprenorphine-naloxone.
- Not consenting to participate to the study.

### Recruitment

#### GPs recruitment

Six academic general medicine departments and six centers for evaluation and information on pharmacodependence-addictovigilance (CEIP-A) will work in pairs to recruit voluntary GPs within their network of working GPs and through regional addiction networks.

Recently, in France, general medicine was recognized as a specialty with a specific training. Consecutively, academic general medicine departments were developed in each faculty. In 2008, an academic pathway of general medicine was created.

In order to assist the French health authorities, in charge of the monitoring and the scheduling of psychoactive substances with abuse potential, a monitoring system for psychoactive medications abuse consisting of a national network of 13 centers for evaluation and information on pharmacodependence-addictovigilance (CEIP-A) was created in 1990 [29, 30].

In both sources for selection, GPs will be contacted by postal mail comprising a questionnaire for validating inclusion criteria for the GP, and an agreement form for participating to the ESUB-MG study. A pre-stamped return envelope will be included. Monetary compensation is planned for participating GPs as compensation for the time and contribution to the study.

GPs in both groups will receive both oral and written information about study design and conduct (patients’ recruitment, inclusion criteria). Material for data collection will comprise a GP questionnaire on basic demographic information and location, GP information notice (detail on recruitment and study conduct), questionnaire for the inclusion and follow up visit.

GPs agreeing to participate to the study will be randomly assigned to one of two groups: one group with intervention, one control group.

#### Patients’ recruitment

GPs will be requested to include all consecutive patients that would be eligible. Each GP have to include at least two patients within 18 months.

Participant recruitment will commence in March 2016, and patient participation will be completed by August 2018. The planned end date for the trial is December 2019.

### Consent

GPs will inform eligible patients of their involvement in the study and that their medical data will be used for the purpose of the research. As a research on standard care GPs will have to ensure that patient are not opposed to take part to the research.

Patients that would explicitly express their opposition will not be included. Patients who are not opposed to participate but refusing to submit to one or several OS-UDS will be maintained in their group defined by their cluster.

### Randomization

Randomization will be undertaken at the cluster (GP) level. Based on the procedure allocated to GP, all patients within a cluster will be assigned to either intervention or control group.

Randomization of participating GP will be performed after GP’s approval and collecting complete questionnaire including basic demographic information and their location. The randomization list will be generated by an independent biostatistician in the clinical research methodological support unit (Unité de Soutien Méthodologique à la Recherche Clinique USMR) of the University hospital (Centre Hospitalier Universitaire CHU) of Toulouse, France. Clusters will not be randomized all at once (first patients inclusion need to begin whereas GPs’ recruitment will be on-going), thus the allocation for each consecutive participating GP will be obtained from the USMR through a specific website. To avoid contamination bias, no more than one GP could be included in a given medical practice.

### Intervention

Intervention will consist in: 1) a training session for GPs on use and interpretation of OS-UDS; 2) the supply of OS-UDS at GPs’ medical offices; 3) performing an OS-UDS before the first prescription of buprenorphine. GPs will be let free to perform OS-UDS for the follow-up if they judge it necessary.

GP assigned to the intervention group will be visited by a clinical research assistant (CRA) to be trained on the methods for performing test (urinary sample collection and reading of the test results). The training session is expected to last about 1 h, and a written guidance will be provided. Material for testing (OS-UDS) will also be supplied during this session. OS-UDSs will be centrally bought by the CHU de Toulouse and will be provided by the CRA in charge of the training session.

During the consult, GPs of the intervention group will dedicate an average 5 min to perform OS-UDS. Patients will be asked to collect a urine sample at the GP’s medical office. GP will read and communicate the results immediately to the patients. GPs will keep free of their management according to OS-UDS results.

#### OS-UDS characteristics

OS-UDS will be in accordance with positivity threshold recommended by the National Institute on Drug Abuse (NIDA) [31] and the Substance Abuse and Mental Health Administration (SAMSHA) [32]. The SAMSHA increase the threshold for screening opiates in 2008 (2000 ng/mL instead of 300 ng/mL) to avoid false positive to ingestion of poppy-seeds. Nevertheless, many laboratories maintained the threshold of 300 ng/mL to preserve the opiates screening sensibility [33]. Thus, we will use the threshold of 300 ng/mL.

Several substances will be screened through the OS-UDS in our study: buprenorphine, methadone’s metabolite, 2-ethylidine-1,5-dimethyl-3,3 diphenylpyrrolidine (EDDP), opiates, and cocaine. Opiate substitutes should be systematically screened before starting buprenorphine, according to guidelines. Buprenorphine screening is intended to assess adherence to OMT after initiation, whereas opiates are screened to monitor concomitant consumptions over the course of OMT, or to confirm an opiate addiction before the first prescription (which confirms the indication of OMT). Methadone screening is intended to rule out an ongoing treatment by methadone. Cocaine is often consumed with opiates. In France, at least 10 % of patients would be concerned by cocaine consumption while they are treated with an opioid substitute [34, 35], and it is associated with negative outcome. Some patients could request for an OMT being unaware of the indication of these drugs and believing they could be offered such treatment.

Positivity thresholds currently used [31, 32] and that we will use are: buprenorphine: 10 ng/mL ; EDDP (the metabolite of methadone): 100 ng/mL ; opiates: 300 ng/mL ; cocaine: 300 ng/mL.

At these thresholds, sensibility was 80 % (IC 95 %: 55–100) and specificity 99 % [96 – 100] for opiates, sensibility not calculable and specificity 100 % [100–100] for cocaine [19]. In another study, sensibility for buprenorphine with 3 different OS-UDS varied from 88 to 100 % and specificity from 91 to 100 % [36].

#### Controlled group

Controlled arm will correspond to standard medical strategy for assessing consumptions while prescribing OMT. Excluding OS-UDS, there will be no prohibited procedure. In particular, GPs of the controlled harm are authorized to implement any biological test to ascertain associated substances use, including for instance laboratory testing. However, according to previous data, we can expected that few drug tests should be performed in this control group: 1 to 3 % [11, 27].

### Outcome measures

The primary outcome will be OMT retention at 6 months. Secondary outcomes will be patient adherence to buprenorphine, associated psychoactive substances use, acceptability of OS-UDS reported by the patient, acceptability of OS-UDS reported by the GP.

#### Primary outcome

Retention in treatment at 6 months will be the main judgment criterion. Actually, a review on all Cochrane systematic reviews performed by the Cochrane Review Group on Drugs and Alcohol highlighted that the main outcomes used in studies assessing effectiveness of opiate maintenance treatment were retention in treatment and illicit use of heroin [4]. Whatever the treatments compared, the retention in treatment was the most constant and the most reproducible outcome used over the different clinical trials because heroin use (assessed through different ways, self-reported or through urinary analysis) is rarely reported in a standardized way. This outcome could be considered as intermediate steps of treatment for heroin-addicted patients. Because observational studies showed high rates of mortality in heroin-addicted patients [37], especially early after discharge from treatment, the ability of a treatment in retaining people in treatment should be reported as a proxy of effectiveness [38].

Retention in treatment will be defined as patients remaining under opiate maintenance treatment at 6 months in a context of medical care (i.e. drug prescribed by a physician, not diverted or obtained through an illegal way, whatever the drug considered, buprenorphine; buprenorphine/naloxone, methadone) and assessed by the general practitioner at the end of the follow-up. Patients switching from buprenorphine to methadone or to buprenorphine/naloxone during study follow-up will be considered as remaining under opiate maintenance treatment.

The patient will be defined as retained in treatment if he will be prescribed by the same GP a legal opiate maintenance drug, or if the drug will be prescribed by another practitioner in connection with the treating physician. In case of loss of follow-up or diverted use of drug (intravenous or nasal route or illegal acquisition of the OMT drug), the patient will be considered as not retained. In case of death, the patient will be considered as maintained until the date of death, and censored after this date. Buprenorphine must be prescribed under strict conditions for a maximum of 28 days (methadone for a maximum of 14 days). Consequently, a patient not attending a medical visit for more than 56 days (2 months) should be considered as not remaining under opiate maintenance treatment.

Retention in treatment has to be recorded at 6 months (time window tolerated of +/− 14 days) as there is no scheduled or mandatory visit for the patient.

#### Secondary outcomes

The secondary judgment criteria will be the adherence to buprenorphine associated psychoactive substances use, and acceptability of OS-UDS reported by the patient, the GP.

We will specifically collect for both groups: characteristics of buprenorphine utilization (dose, duration), exposure to opiate or other illegal substances (heroin, morphine, cannabis, cocaine, benzodiazepines, amphetamines, other…) during the follow-up (self-reported during medical examination and/or biologically assessed); for the intervention group: number of OS-UDS performed by GP and by patient acceptability reported by the patient and by the GP (self-reported questionnaires).

#### Cross-link data

To ensure completeness in prescription drug records, significant medical event and death occurring during follow up, additional information on medical care will also be obtained through the database of the health insurance scheme. A query into the information system from the national health insurance scheme database (SNIIRAM) and national mortality registry will be done to complete follow-up [39]. Patients will be matched thanks to a probabilistic match [40]. In a recent study, around 80 % of patients had been matched between general practice data and health insurance scheme data [41]. Data collected will not be included in the main analysis.

### Sample size calculation

Comparisons between groups will be performed taking into account clustering which the unity will be the GP. Thus the sample size must be corrected by an inflation factor according to guidelines on clustered analyses [42–45]. According to the results of a previous study performed by our group on patients initiating buprenorphine in ambulatory care in the Midi-Pyrénées area [27], we were able to identify clusters of GPs and calculated the intra-cluster correlation coefficient (CCI = 2.79 %) and the mean cluster size (m = 2), giving an inflation factor (IF = 1.03).

According to the literature, the retention rate with buprenorphine at 6 months is generally around 40 %, with 60 % in specialized addiction centers with urinary testing and supervision [4, 5]. In studies performed in our area, observed retention rates were similar [13]. Using reimbursement data from the French Health Insurance system at the regional level, we compared retention rate of patients newly treated by buprenorphine according to performing or not urinary testing [27]. In this study including 1,507 subjects followed-up over 30 months, the retention rate in patients with urinary testing was significantly better than the reference group, with an adjusted Hazard Ratio of 0.55 (95 % CI: 0.38–0.80).

Thus, considering a retention rate in the reference group of 36 %, and an expected retention rate of 50 % in the intervention group, if mean cluster size (m) = 2, α risk = 0.05 and β risk = 0.20, the theoretical formula of Hayes and Moulton gives 100 clusters by group, i.e. 100 GPs in each group, corresponding to 200 patients in each group, i.e. 400 patients in all [45].

Recruiting 2 patients by GPs over a period of 18 months seems realistic, even if these GPs are not working in an addiction specialized network. Hypothesizing that the expected retention rate will be 50 % in the intervention group is very conservative. Actually, with an expected retention rate of 60 % (as observed in our previous study) or with an expected relative risk of 0.3-0.4 in favour of the intervention group (as observed in the McCowan study [28], with a benefit of performing urinary testing -whatever the results- on mortality in patients treated by methadone in UK). With this retention rate, only 34 clusters should be needed, corresponding to 34 GPs in each group, 68 in the all sample, and consecutively 136 patients. This hypothesis should be probably optimistic but not completely unrealistic.

This strategy will allow to overcome the proper effect of each GP, to get enough clusters for the analysis and to regroup clusters (on the basis of the same geographic area (in French “Bassins de santé”) giving a sufficient number of individuals in each cluster (5 subjects).

**Fig. 1:**
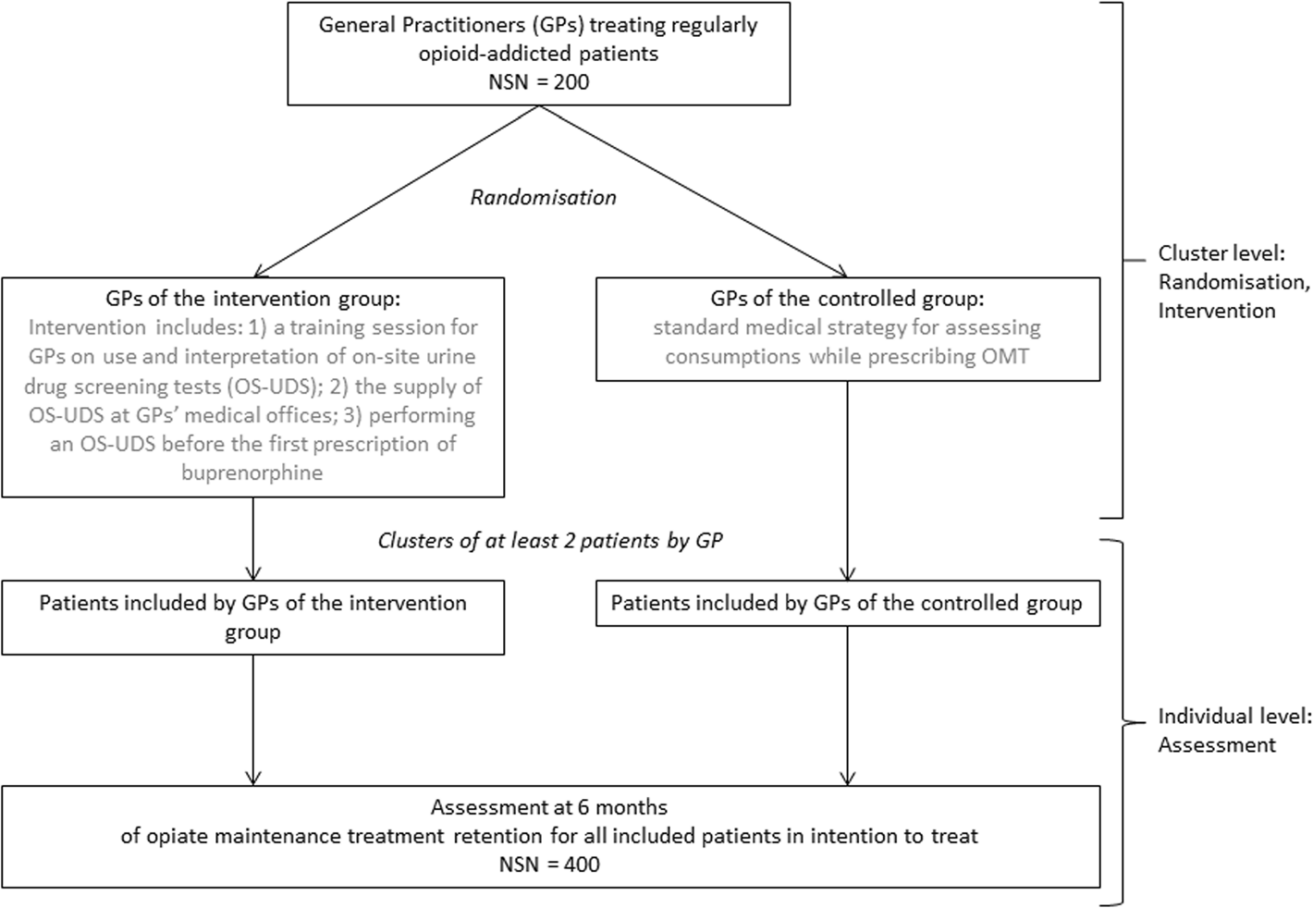
Design of the ESUB-MG pragmatic randomized controlled trial

The Fig. 1 summarizes the design of the study.

### Statistical analysis

Patients, will be analyzed according to the intervention assigned to their GPs, whether being exposed to OS-UDS during their participation or not, in accordance with the intention-to-treat principle.

For patients followed up until the end of the study, the retention rate will be the percentage of patients still under OMT (complete data). For loss of follow up patients, an intention to treat approach will be used and loss of follow will be analyzed as failure.

A description of the baseline characteristics of the group will be performed, comprising mean ± SD for continuous variables and frequency and percentages for qualitative variables. Baseline characteristics and secondary outcomes will be compared among groups using the Chi2 test of independency or Fisher test for categorical variables and the Student t test or the Wilcoxon test for continuous variables. A significance threshold of 5 % will be applied for all the statistical analyses.

Retention at 6 months will be computed as an individual binary variable, and will be analyzed using mixed effect logistic regression, including GPs as a random effect parameter. Potential confounders or explanatory variables at individual and cluster level will be included. An alternative way for analysis would consist in applying generalized estimating equation (GEE). These models are appropriate in the study design as the number of cluster will be higher than 15 [43].

Univariate analyses on baseline variables as potential predictors for success or failure of OMT maintenance will be performed using Chi-square statistics for categorical and Student t-test for continuous data. Variables with a P-value of <0.2 after univariate analysis will be entered into a multivariate logistic regression model. Crude and adjusted Odds Ratio (OR) and their confidence intervals will be estimated. The main analysis will be completed by univariate and multivariate clustered survival analyses.

Analyses will be performed using the SAS ® 9.3 software (SAS Institute Inc., Cary, NC, USA).

### Ethical approval

The study will be done in the French regulatory context of standard care (in French “Soins Courants”), all the procedures used in the study being in the standard care of opiate addicts. Thus, the study will not modify the standard follow-up of patients newly treated by an OMT and all the medical visits and/or other interventions will be done as needed.

This study has been approved by Persons’ Protection Committee (CPP) of Bordeaux, France (n°2014-A00393-44) and the Consultative Committee on Data Processing in Research in the Area of Health (CCTIRS) (n°14.356bis). Data treatment was authorized by the National Commission for Computing and Civil Liberties (CNIL) (application n°915030, decision DR-2015-112).

The French Ministry of Health funded the project after peer-reviewing the protocol. The body organization had no role in the writing of the manuscript.

## Discussion

This randomized intervention trial in primary care context should bring sufficient level of evidence to assess effectiveness of performing OS-UDS in general practice for patients treated by buprenorphine. The aim is to assess the impact of a global intervention, including a better knowledge of UDS (through a specific GP’s training to perform UDS and interpret their results), and giving the opportunity to perform OS-UDS for any patient consulting for an OMT initiation in the medical office by the GP. The better way to assess this impact with a sufficient level of evidence is to perform a randomized intervention trial in primary care context, comparing OMT retention at 6 months in patients cared by GPs randomly assigned to having on-site UDS, compared to patients cared by GPs randomly assigned to performing standard care.

Most of OMT patients in France are managed in the context of primary care, whereas most of OMT clinical assessments have been done in the context of specialized centers. UDS should be used in this context of primary care, but are rarely done. Commercial kits are giving the possibility to perform UDS extemporarily in the medical office. The limits of these tests in terms of sensitivity and specificity are well-known, and this project does not aim to assess the intrinsic validity of these tests, assuming that they present a sufficient quality to be licensed in France, but to assess effectiveness of performing OS-UDS in general practice for patients treated by buprenorphine.

The widespread use of UDS is already a reality for some (few) GPs working in specialized addiction networks and in centers without laboratories inside. However, outside this context, urine testing is rare and knowledge of GPs remains scarce. Demonstrating the positive impact of OS-UDS on GPs’ practice (in managing patients) and behaviors of patients treated with buprenorphine in general practice (adherence) would be an important issue in the field of opioid addiction care.

Training GPs to drug tests and supplying OS-UDS in their office should lead to an improvement of opioid-addicted patients’ care through helping decision making in the GP medical office, improving GPs’ practices, improving adherence of treated patients, and consequently, improving short and long term outcomes of OMT.

## Trial status

Inclusions of GPs and patients will begin from January 2016 to March 2016 according to French regions. At the time of manuscript submission, the ESUB-MG study is ready to include GPs and patients. GPs’ inclusions are expected to continue until August 2016. Patients’ inclusions are expected to continue until January 2018. Data collection is expected to continue until July 2018.

## Abbreviations

CEIP-A: centers for evaluation and information on pharmacodependence-addictovigilance
CHU: Centre Hospitalier Universitaire (University hospital)
CRA: clinical research assistant
EDDP: 2-ethylidine-1,5-dimethyl-3,3 diphenylpyrrolidine
GPs: general practitioners
NIDA: National Institute on Drug Abuse
OMT: opiate maintenance treatment
OS-UDS: on-site urine drug screening
SAMSHA: Substance Abuse and Mental Health Administration
SNIIRAM: information system from the national health insurance scheme database
UDT: urine drug screening test
USMR: Unité de Soutien Méthodologique à la Recherche Clinique (clinical research methodological support unit)
